# Calcium and Magnesium Regulation of Kernel Sugar Content in Maize: Role of Endogenous Hormones and Antioxidant Enzymes

**DOI:** 10.3390/ijms26010200

**Published:** 2024-12-29

**Authors:** Zhaoquan He, Xue Shang, Xiaoze Jin, Xiukang Wang, Yingying Xing

**Affiliations:** 1 School of Life Sciences, Yan’an University, Yan’an 716000, China; 2 Shaanxi Key Laboratory of Research and Utilization of Resource Plants on the Loess Plateau, College of Life Sciences, Yan’an University, Yan’an 716000, China; 3 Shaanxi Key Laboratory of Chinese Jujube, Yan’an University, Yan’an 716000, China; 4 College of Land Resource and Environment, Jiangxi Agricultural University, Nanchang 330045, China

**Keywords:** Ca and Mg supplementation, endogenous hormones, antioxidant enzymes, kernel sugar content, maize

## Abstract

Ca and Mg are essential micronutrients for plant growth, and they play a crucial role in plant development and responses to adversity by influencing the activities of endogenous hormones and antioxidant enzymes. However, the specific mechanisms through which calcium (Ca) and magnesium (Mg) regulate the kernel sugar content through endogenous hormones and antioxidant enzymes remain unclear. In this study, we analyzed the impact of Ca and Mg on the physiology of maize leaves and kernel quality by determining the activities of antioxidant enzymes and endogenous hormones, and the kernel sugar content in maize leaves when supplemented with different levels of Ca and Mg. Our main findings were as follows: (1) Elevated Mg levels augmented superoxide dismutase (SOD) activity, bolstering antioxidant defenses, whereas low Ca and Mg levels diminished SOD activity. High Ca levels enhanced catalase (CAT) activity during kernel development. Low−Ca conditions stimulated gibberellin (GA) synthesis, while high−Ca and high−Mg conditions suppressed it. High Mg levels also elevated abscisic acid (ABA) levels, potentially improving stress tolerance. (2) High Ca levels increased the reducing sugar content in kernels, augmenting the energy supply, while both low and high Mg levels increased soluble sugars, with low Mg levels specifically enhancing the sucrose content, which is a critical energy reserve in plants. (3) CAT exerted a pivotal regulatory role in the sugar accumulation in maize kernels. GA, under the influence of Ca, modulated the sucrose and soluble sugar contents by inhibiting CAT, whereas ABA, under the influence of Mg, promoted CAT activity, thereby affecting the kernel sugar content. This study reveals a new mechanism through which the addition of Ca and Mg regulate the sugar content in maize kernels by affecting endogenous hormones and antioxidant enzyme activities. These findings not only enhance our understanding of the role of micronutrients in plant growth and development but also provide new strategies for improving crop yield and stress tolerance.

## 1. Introduction

Maize (*Zea mays* L.) is a globally significant food crop, and the kernel sugar content is a pivotal determinant of its nutritional quality and directly impacts its eating and processing qualities [1]. Research on maize sugar contents can be traced back to the early 1900s, when scientists began investigating the effects of endogenous plant hormones and mineral elements on crop quality [2]. Calcium (Ca) and magnesium (Mg), as essential mineral elements for plant growth and development, play crucial roles in physiological processes such as photosynthesis, respiration, protein synthesis, and starch accumulation. The influence of Ca and Mg fertilizer application on sugar transport and partitioning in maize kernels was first demonstrated in the mid−20th century [3].

As the research advanced, the effects of Ca and Mg on endogenous hormone levels in maize leaves were increasingly recognized. Studies have shown that Ca and Mg fertilizers exert a synergistic effect on endogenous hormone levels in maize leaves, influencing various physiological and biochemical processes [4]. These include the regulation of the levels of the hormones indole−3−acetic acid (IAA), gibberellin (GA), and zeaxanthin riboside (ZR), as well as the activities of enzymes and protein synthesis pathways [5]. These findings provide a theoretical foundation for the application of Ca and Mg fertilizers to increase the sugar content in maize kernels.

Previous studies have focused on the effects of Ca and Mg fertilizers on maize growth and development, and how these mineral elements regulate plant growth by affecting endogenous hormone levels [6,7]. These studies demonstrated that moderate applications of Ca and Mg fertilizers can promote the transport of sucrose from leaves to kernels and enhance the synthesis and accumulation of sucrose in kernels during the filling period [8]. Furthermore, the synergistic effect of Ca and Mg on hormone levels leads to improved nutrient growth indices, such as plant height, leaf area, and chlorophyll concentration. The improvements in these growth parameters and quality indices are attributed to the nutrient uptake facilitated by these fertilizers [9].

The impact of Ca and Mg fertilizers on antioxidant enzyme activities in maize leaves has been a prominent research topic, particularly in the context of antioxidant enzyme activities [10]. It has been observed that elevated hormone levels, resulting from Ca and Mg supplementation, are associated with increased antioxidant enzyme activities. These elevated activities aid in mitigating oxidative stress, thereby supporting the growth and development of maize [11,12]. The synergistic effect of Ca and Mg enhances the activities of antioxidant enzymes in maize kernels [13]. This synergistic effect may enhance resistance to oxidative damage and support overall plant growth.

Although previous studies have shown the effects of Ca and Mg fertilizers on the sugar content levels in maize kernels, there remains a divergence in viewpoints, theories, and conclusions. Some studies emphasized the direct role of Ca and Mg fertilizers in promoting the sugar content in maize kernels, while others have focused on their indirect regulation of the sugar content through the influence on endogenous hormones and antioxidant enzyme activities [14,15]. For instance, one study noted that the combined application of Ca and Mg fertilizers increased the levels of these nutrients in maize kernels, which was positively correlated with enhanced antioxidant enzyme activities. In contrast, other studies have highlighted the effects of applying either Ca or Mg fertilizers [16].

The differences in the perspectives of these studies may have arisen from the different experimental conditions, maize varieties, or environmental factors. However, the general consensus is that Ca and Mg fertilizers positively affect the sugar content of maize kernels [17]. Nonetheless, these studies are limited in that they often overlook the balance between Ca and Mg and the potential negative impacts of this balance on plant growth and sugar content. Additionally, they typically fail to explore how Ca and Mg fertilizers regulate the sugar content by influencing endogenous hormones and antioxidant enzyme activities, which is a significant area of research that requires further elucidation [18,19].

In summary, while previous studies have yielded certain results, there remain research gaps and shortcomings. Firstly, the specific mechanisms through which Ca and Mg fertilizers regulate the sugar content of maize kernels through their effects on endogenous hormones and antioxidant enzyme activities remain poorly understood. Secondly, the effects of the Ca−to−Mg balance on crop growth and sugar content have not been thoroughly investigated. Furthermore, most studies have concentrated on laboratory conditions, and the effects and impacts of Ca and Mg fertilizers under actual agricultural production conditions have yet to be clarified [20].

Therefore, this study investigated the effects of varying levels of Ca and Mg supplementation to maize in the dry−crop region of northern Shaanxi Province, with the intent to achieve the following objectives: (1) to analyze the dynamics of the changes in endogenous hormones and antioxidant enzymes in maize leaves, as well as the characteristics of the changes in the sugar content of maize kernels across different stages of maturation, and the interrelated response processes, and (2) to elucidate how Ca and Mg fertilizers regulate the sugar content levels in maize by influencing the activities of endogenous hormones and antioxidant enzymes, thereby revealing the physiological mechanisms underlying the regulation of the maize kernel sugar content. Through an in-depth examination of this regulatory mechanism, we aim to provide more scientifically informed fertilization strategies for maize cultivation and quality enhancement. This study is not only crucial for advancing the theoretical understanding of crop nutritional physiology but also holds potential implications for agricultural practices and crop breeding strategies.

## 2. Results 

### 2.1. Changes in Endogenous Hormones and Antioxidant Enzymes in Maize Leaves

**Figure 1:**
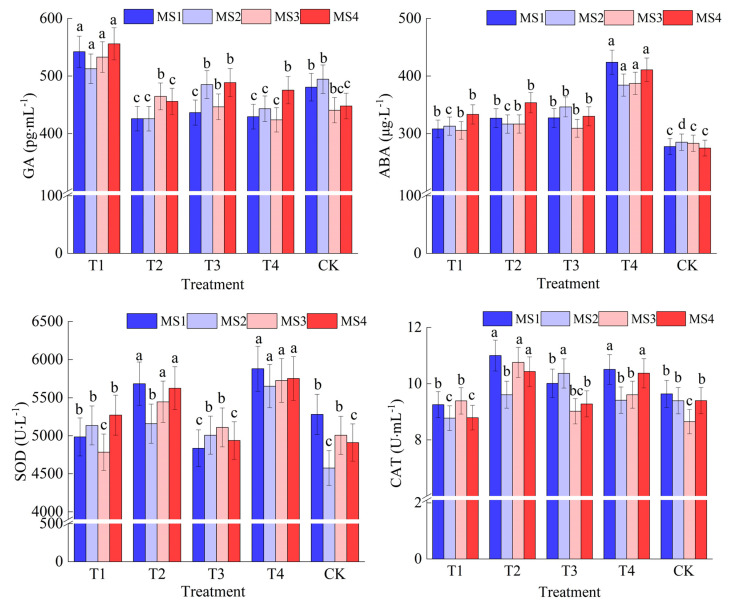
Changes in endogenous hormone content and antioxidant enzyme activity in maize leaves under calcium and magnesium supplementation. The line on each column represents the standard deviation (SD). SOD, CAT, ABA, and GA represent superoxide dismutase, catalase, abscisic acid, and gibberellin, respectively (the same below). MS1, MS2, MS3, and MS4 represent the 10th, 20th, 30th, and 40th days of the milk ripening period of maize (the same below). a, b, c, and d represent the differences in the same physiological index of the different treatments at the same time point at the level of *p* < 0.05.

Regarding the mean superoxide dismutase (SOD) values, that of T1 was lower than that of CK, suggesting that the SOD activities of the T1 treatment were generally lower from the milk ripening to full maturity stage. The T2 value was higher than that of CK, indicating that the SOD activities of the T2 treatment were generally higher from the milk ripening to the full maturity stage. The T4 value was significantly higher than that of CK (*p* < 0.05), indicating that the SOD activities of the T4 treatment were significantly higher from the milk ripening to full maturity stage. From a temporal perspective, the activity of T1 peaked on day 20 and then gradually decreased, while the activity of T2 peaked on day 30 and then slightly decreased. The activity of T3 was highest on day 30, which then gradually decreased. The activity of T4 continued to increase until it reached its peak on day 40, whereas the activity of CK remained the lowest from day 20 onwards (Figure 1). It can be concluded that the T4 treatment had the highest SOD activity at all time points, which suggests that the T4 treatment was the most effective in enhancing the antioxidant capacity of maize leaves. The generally lower SOD activity of the T1 and T3 treatments may indicate that these treatments were less effective in increasing the antioxidant capacity. The T2 treatment had the lowest SOD activity on day 20 and it continued to increase thereafter, which suggests that this treatment was particularly effective at the mid to later stages of the season (Figure 1).

The mean catalase (CAT) values for treatments T1 and CK were very similar, suggesting that the effect of the T1 treatment on CAT activity was comparable to that of the control. The mean values of T2 were significantly higher than those of CK (*p* < 0.05), indicating that the T2 treatment significantly increased CAT activity. The mean values of T3 and T4 were also higher than those of CK, but the enhancement was not as significant as that observed in T2. Regarding the time course, T1 and CK exhibited a similar trend, with the activity peaking on day 10 and then gradually decreasing. T2 reached its highest activity on day 10, demonstrating a decreasing trend over time. T3 and T4 displayed a fluctuating trend; T3 peaked on day 20 and then decreased, while T4 reached its lowest point on day 20 and then increased (Figure 1). It was evident that the T2 treatment showed the best performance in increasing CAT activity, and its average activity was significantly higher than that of the control from the milk ripening to full ripening stage. T1 and CK showed similar performances, indicating that the T1 treatment did not have a significant effect on CAT enzyme activity. Although T3 and T4 showed higher activity at some points during ripening, their overall impact was not as significant as that of T2.

The average GA content in the T1 treatment was higher than that of CK, suggesting that the T1 treatment may have promoted GA synthesis or release. The average GA content in the T2 treatment was lower than that of CK, suggesting that the T2 treatment may have inhibited GA synthesis or release. The average GA content of both the T3 and T4 treatments was lower than that of CK; however, the effect of T3 was closer to that of CK, while the effect of T4 was similar to that of T2, suggesting that these treatments may have had some influence on GA synthesis or release to varying extents. In the time series, the T1 treatment reached its highest GA content on day 40, suggesting that this treatment may promote GA synthesis in the late ripening stage. The GA content of the T2 treatment peaked on day 30 and then declined, suggesting that this treatment may promote GA synthesis in the early ripening stage, but the effect diminished thereafter. The GA content of the T3 treatment peaked on day 20 and then slightly declined, suggesting that this treatment promoted GA synthesis from the milk ripening to the full ripening stage. The GA content of the T4 treatment was lower on day 10 and then gradually increased, suggesting that this treatment may promote GA synthesis in the later part of the ripening stage (Figure 1). Thus, it can be concluded that the T1 treatment promoted the synthesis or release of GA, particularly at the later stage of maturity. The T2 and T4 treatments inhibited the synthesis or release of GA, particularly at the early stage of maturity. The effect of the T3 treatment was intermediate between that of T1 and T2, suggesting that the treatment may have some promotion of GA synthesis, but not as pronounced as that of the T1 treatment.

The average ABA contents of treatments T1, T2, and T3 were lower than that of T4, and the ABA content of T4 was significantly higher than that of CK (*p* < 0.05), indicating that the T4 treatment significantly promoted the synthesis or release of ABA. The average ABA contents of treatments T1, T2, and T3 were similar to those of the control CK, suggesting that these treatments had minimal impact on the synthesis or release of ABA. In the time series, the ABA content of the T1 treatment peaked on day 40, suggesting that this treatment may promote ABA synthesis during the late ripening period. The ABA content of the T2 treatment also peaked on day 40, showing an increasing trend over time. The ABA content of the T3 treatment was highest on day 20 and then slightly decreased, indicating a beneficial effect on ABA throughout the milky ripening to full ripening period. The ABA content of the T4 treatment was higher at all time points from the milk ripening to full ripening stage and reached its maximum on day 10, suggesting that this treatment may continuously promote ABA synthesis throughout the ripening period (Figure 1). The above analysis indicates that the T4 treatment significantly increased the ABA content in maize leaves, which may suggest that the T4 treatment had a positive effect on improving plant stress tolerance. The ABA contents of treatments T1, T2, and T3 were similar to that of the control, suggesting that these treatments did not significantly affect the synthesis or release of ABA.

### 2.2. Variation in and Correlation of Sugar Content in Maize Kernels

**Figure 2:**
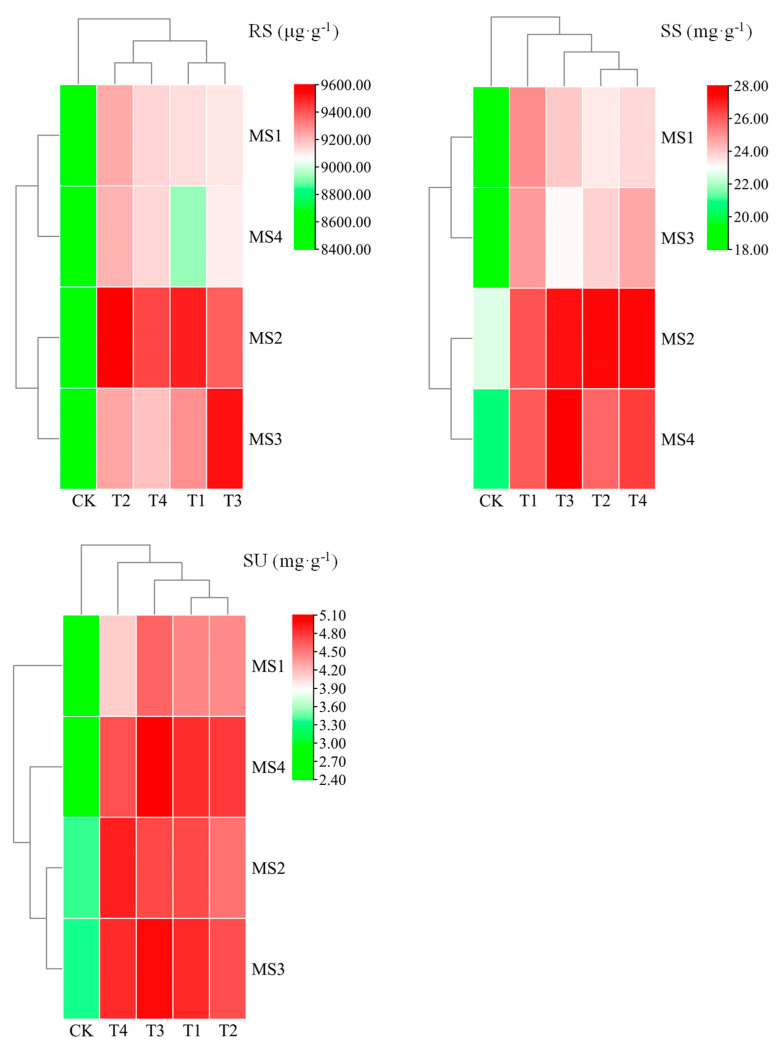
Changes in sugar content in maize kernels under supplementation with calcium and magnesium. RS, SS, and SU represent reducing sugars, soluble sugars, and sucrose, respectively (the same below).

The mean reducing sugars (RS) content across all the treatments (T1 to T4) was found to be higher than that of CK, suggesting that these treatments may have facilitated RS synthesis or release. Among these, the T2 treatment exhibited the highest mean RS content, indicating that it was the most effective in promoting RS synthesis or release. Regarding the time series data, the RS content for the T1 treatment reached its maximum on day 20 and then declined gradually. The RS content for the T2 treatment peaked on day 20 and subsequently experienced a slight decline. Similarly, the T3 treatment’s RS content reached its zenith on day 30, followed by a slight decrease. The T4 treatment mirrored this pattern, with the RS content reaching its maximum on day 20 and then decreasing slightly. In summary, the T2 treatment significantly elevated the RS content in maize kernels, potentially indicating a positive impact on enhancing the plant’s energy supply. While the RS contents in the T1, T3, and T4 treatments were also higher than that of the control, these increases were not as pronounced as in the T2 treatment (Figure 2).

The mean soluble sugars (SS) content across all the treatments (T1 to T4) was higher than that of CK, suggesting that these treatments may have promoted the synthesis or release of SS. The mean SS contents of treatments T1, T3, and T4 were similar and higher than that of CK, while the mean SS content of the T2 treatment was slightly lower than those of T1, T3, and T4, yet still higher than that of CK. Additionally, the SS content for all treatments (T1 to T4) peaked on day 20, after which, there was a slight decrease (Figure 2). These results indicate that treatments T1, T3, and T4 significantly increased the SS content in maize kernels, potentially enhancing the plant’s energy supply. Although the SS content of the T2 treatment was also higher than that of the control, the increase was not as pronounced as those of treatments T1, T3, and T4.

The mean sucrose (SU) content across all the treatments (T1 to T4) was higher than that of CK, suggesting that these treatments may have promoted the synthesis or release of SU. Notably, the T3 treatment exhibited the highest mean SU content, indicating that it was the most effective in promoting SU synthesis or release. In the time series analysis, the T1 treatment displayed the highest SU content on day 40, suggesting that this treatment may facilitate SU synthesis during the later stages of maturity. Both the T2 and T3 treatments reached their peak SU content on day 30, after which, there was a slight decrease. The T4 treatment peaked in SU content on day 20, followed by a slight decrease. In conclusion, the T3 treatment significantly increased the SU content in maize kernels, which may suggest that it has a positive effect on enhancing the energy supply of maize. Although the SU contents of the T1, T2, and T4 treatments were also higher than that of the control, the increase was not as pronounced as that observed in the T3 treatment (Figure 2).

**Figure 3:**
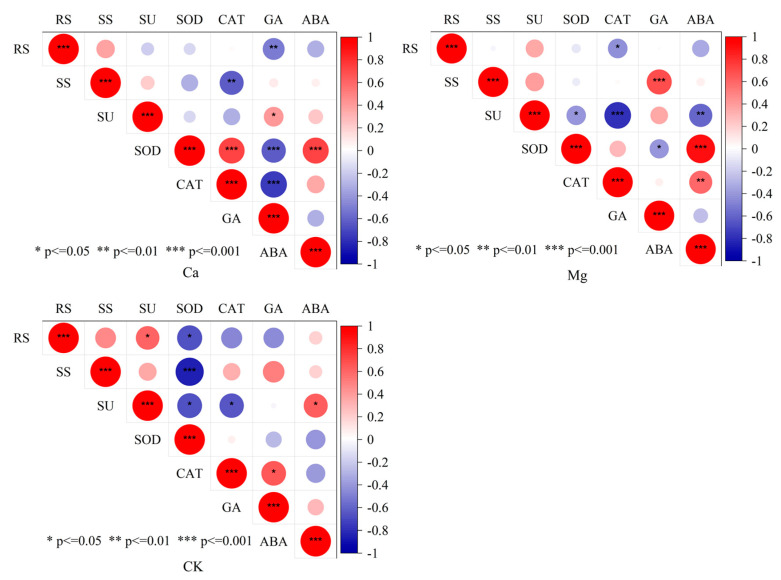
Correlations between maize kernel sugar content and leaf endogenous hormones and antioxidant enzymes. * *p* ≤ 0.05, ** *p* ≤ 0.01, and *** *p* ≤ 0.001 represent the correlations that reached significant and extremely significant levels.

The correlation analysis revealed that after Ca supplementation, the maize leaf SOD activity exhibited a highly significant positive correlation with the CAT activity and ABA content (*p* < 0.001). Conversely, the GA content showed a highly significant negative correlation with the RS content, and SOD and CAT activities, and the kernel SS content also demonstrated a highly significant negative correlation with the leaf CAT activity (*p* < 0.01) (Figure 3). Following Mg supplementation, the kernel SU content displayed a highly significant negative correlation (*p* < 0.001) with the leaf CAT activity and ABA content, while the kernel SS content exhibited a highly significant positive correlation (*p* < 0.001) with the leaf GA content. Additionally, the leaf SOD activity showed a highly significant positive correlation (*p* < 0.001) with the ABA content. In the control treatment, the kernel RS, SS, and SU contents revealed significant negative correlations with the leaf SOD activity, whereas the SU content showed a significant positive correlation with the ABA content (*p* < 0.05) (Figure 3).

### 2.3. Physiological Driving Mechanism of Sugar Content Accumulation in Maize Kernels

**Figure 4:**
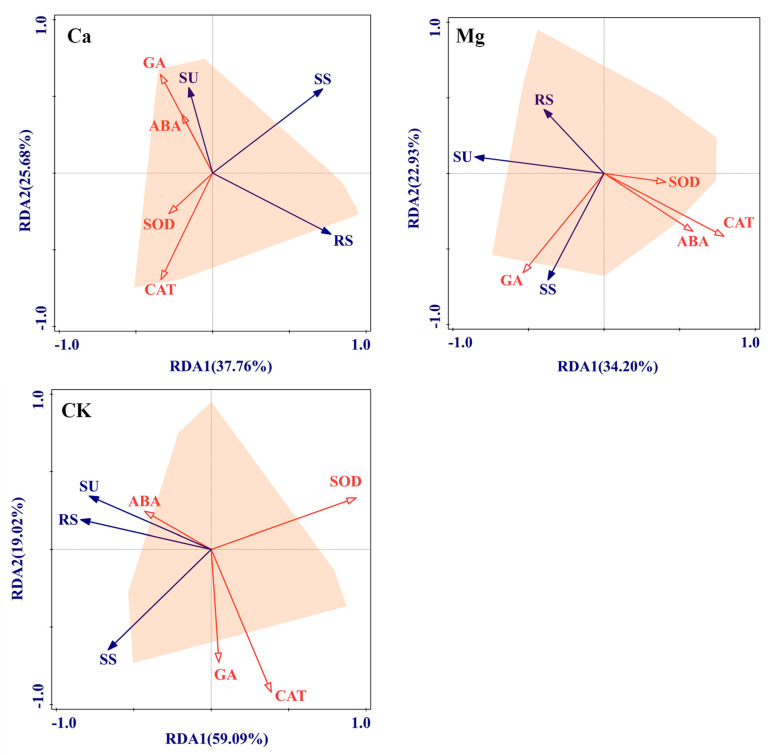
Redundancy analysis of physiological driving factors for sugar content accumulation in maize kernels under calcium and magnesium supplementation.

The redundancy analysis revealed that the kernel’s SU content was primarily positively regulated by the leaf GA content and negatively regulated by the leaf CAT activity following the addition of Ca. In contrast, the kernel’s SS content was predominantly negatively regulated by the leaf CAT activity. The principal leaf endogenous hormones and antioxidant enzyme types that regulate the kernel RS content were found to be similar to those affecting the SU content, namely the GA content and CAT activity, but with opposite regulatory effects on RS. After Mg supplementation, the leaf GA content demonstrated a highly significant positive regulation on both the kernel SU and SS contents, whereas the leaf ABA content and CAT activity exhibited highly significant negative regulatory effects on both the kernel RS and SU contents (Figure 4). In the control treatment, the leaf CAT and SOD activities were identified as the core factors regulating the kernel SU and RS contents, both displaying highly significant negative regulatory effects. Furthermore, the leaf GA content and CAT activity had significant positive regulatory effects on the kernel SS content, while the SOD activity had a highly significant negative regulatory effect on the SS content (Figure 4).

**Figure 5:**
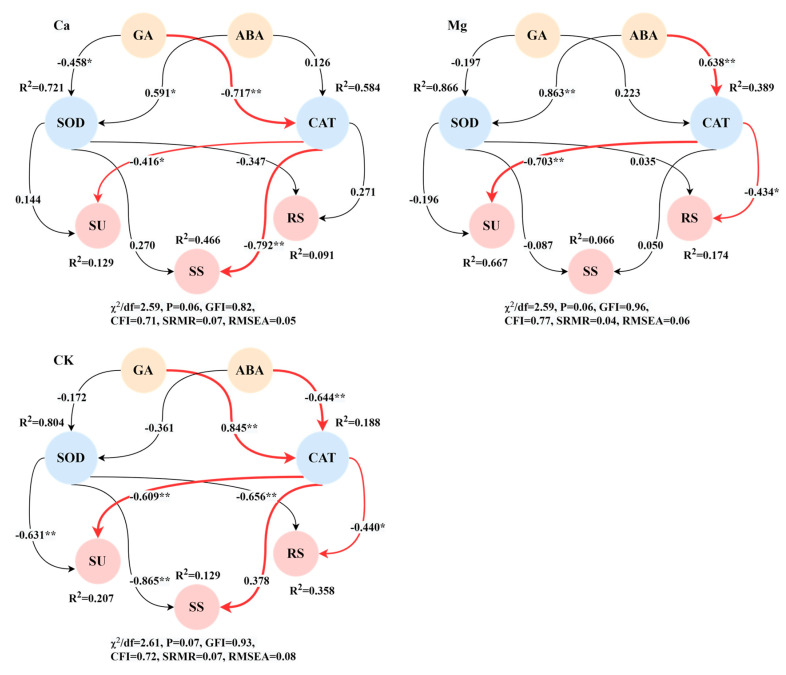
Structural equation model of physiological pathways driving sugar content accumulation in maize kernels under calcium and magnesium supplementation. * represents *p* < 0.05, ** represents *p* < 0.01; χ^2^ represents the chi-square; df represents the degree of freedom; GFI represents the goodness of fit index; CFI represents the comparative fit index; RMSEA represents the root mean square error of approximation.

The structural equation modeling revealed that the core physiological pathways driving the maize kernel sugar content after the addition of Ca were “GA−CAT−SU and SS”. Initially, the GA content negatively regulated CAT activity, with the path coefficient reaching a highly significant level. Subsequently, CAT activity significantly negatively regulated the SU and SS contents. Following the addition of Mg, the core physiological pathway regulating the maize kernel sugar content was “ABA−CAT−SU and SS” (Figure 5). Here, the abscisic acid (ABA) content significantly positively regulated CAT activity, which in turn significantly negatively regulated the SU and SS contents. In the control treatments, the core physiological pathway regulating the maize kernel sugar content was also mediated by endogenous hormones affecting CAT activity; however, the positive and negative effects of the GA and ABA contents on CAT regulation were opposite to those observed with Ca and Mg supplementation (Figure 5).

This indicated that CAT was the central antioxidant enzyme regulating the accumulation of maize kernel sugars, irrespective of Ca and Mg supplementation. Its activity exhibited a direct and significant negative regulatory effect on the accumulation of maize kernel sugars. After the addition of Ca, GA emerged as the core endogenous hormone regulating the maize kernel sugar content, whereas after the addition of Mg, ABA assumed this role.

**Figure 6:**
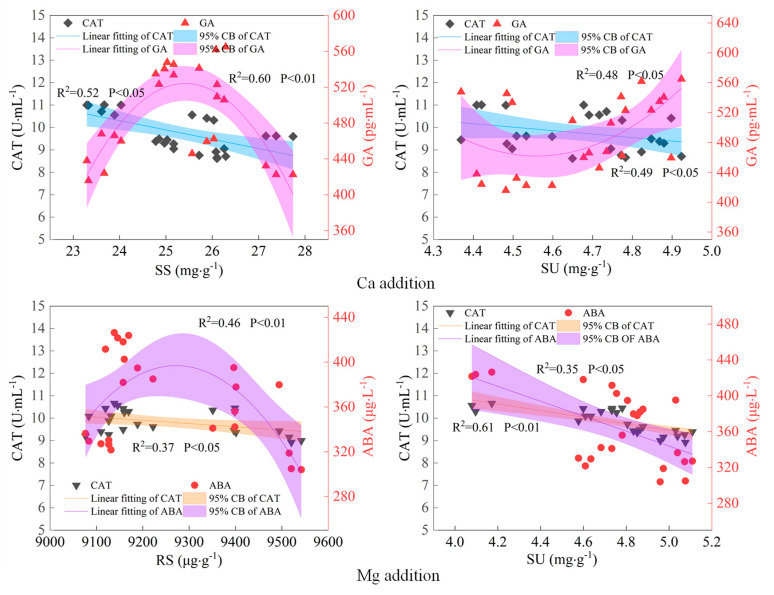
Scatter trend fitting of the sugar content of maize kernels and core physiological driving factors under calcium and magnesium supplementation. CB represents the confidence band.

After establishing the trend relationship between the fitted maize kernel sugar content and its core regulatory physiological indices, it was observed that the addition of Ca led to a negative linear correlation between the kernel SS/SU contents and leaf CAT activity, as well as a nonlinear relationship with the GA content. Following the addition of Mg, the kernel RS content exhibited a negative linear relationship with the CAT activity and a nonlinear relationship with the ABA content; the kernel SU content demonstrated a negative linear relationship with the ratio of the CAT activity to ABA content (Figure 6). These findings underscore the complexity of the relationship between endogenous hormones and the maize kernel sugar content after supplementation with Ca and Mg. The relationship is not merely linear but exhibits a quadratic nonlinear pattern.

## 3. Discussion

### 3.1. Effects of Ca and Mg Supplementation on Endogenous Hormones and Antioxidant Enzymes in Maize Leaves

Our study reveals that Ca and Mg regulate the sugar content in maize kernels by modulating endogenous hormones and antioxidant enzymes. The high−Mg treatments enhanced SOD activity in leaves, likely due to Mg’s role as a cofactor for numerous enzymes, including SOD, which is involved in scavenging reactive oxygen species, thereby boosting the plant’s antioxidant capacity [21]. The high−Ca treatments increased CAT activity from the milk ripening to the ripening stage, possibly because Ca ions play a crucial role in plant signaling, particularly in response to environmental stresses, and in regulating the activities of antioxidant enzymes. Furthermore, the interaction between Ca and Mg significantly affects the synthesis of phytohormones such as GA and ABA, which may be intricately linked to plant growth, development, and stress tolerance [22]. In conclusion, our results suggest that the antioxidant capacity and stress tolerance of maize can be enhanced by modulating the levels of Ca and Mg, which has practical implications for improving the yield and quality of maize under adverse conditions. Notably, in enhancing the nutritional quality of maize, the addition of Ca and Mg may indirectly regulate the sugar content in maize kernels by affecting the activities of endogenous hormones and antioxidant enzymes, potentially improving the nutritional value of the maize [23].

Compared to the existing literature, our study offers new perspectives on crop quality regulation. While Ca and Mg have been shown to stimulate protein synthesis, this study specifically focused on their regulation of the sugar content, which is crucial for understanding the intricacies of crop nutritional quality [24]. Additionally, this study sheds light on the effects of Ca and Mg on antioxidant enzyme activity, an area that had been previously overlooked. However, this study, while providing valuable insights, has some limitations. For instance, it lacked an examination of the long-term effects of Ca and Mg under various environmental conditions and did not control for other potential factors, such as soil pH and temperature [25]. Future studies should explore the dynamics of Ca and Mg under different environmental stresses and their interactions with other nutrients.

### 3.2. Effect of Ca and Mg Supplementation on the Sugar Content of Maize Kernels

In our analysis of the regulatory effects of Ca and Mg on the sugar content of maize kernels, we found that the high−Ca treatments significantly enhanced the reduced sugar content. This enhancement is primarily attributed to the ability of high−Ca treatments to stabilize plant cell membranes and promote cellular metabolic activities, thereby improving the efficiency of sugar synthesis [26]. Additionally, Ca ions, as signaling molecules, may play a regulatory role in the synthesis and release of endogenous hormones, further promoting the adaptive response of crops to environmental conditions.

In contrast, both the low−and high−Mg treatments were particularly effective in increasing the soluble sugar content, potentially due to Mg’s crucial role in photosynthesis and enzymatic reactions [27]. Mg supplementation facilitates the translocation of photosynthetic products, leading to an accumulation of soluble sugars. Interestingly, the low-Mg treatments were notably effective in increasing the sucrose content, a phenomenon that may be associated with the plant stress response induced by Mg iron deficiency, which in turn contributes to sucrose synthesis and storage [28].

The regulatory effects of Ca and Mg not only have the potential to enhance the sugar quality of maize kernels but may also improve the nutritional value and economic efficiency of maize. Amidst growing concerns over global food security, studying the nutritional quality of crops is of paramount importance [29]. Maize kernels with higher sweetness have greater market value in the food and feed production industries; thus, understanding the impact of Ca and Mg on sugar synthesis provides a theoretical foundation and reference for the optimization of maize breeding and cultivation techniques.

Comparing our findings with existing literature, we noted that some results aligned with previous studies, such as the consensus that high−Ca treatments are an effective measure for increasing crop sugars. However, discrepancies in the effects of Mg treatments may arise from differences in experimental conditions and materials, indicating a new direction for future research [30]. Nevertheless, our study has certain limitations, primarily concerning the experimental scale and the homogeneity of environmental conditions, which prevent the broad applicability of our conclusions. Therefore, in subsequent studies, a variety of experimental conditions, including different soil types, climatic conditions, and maize varieties, should be considered to further validate the role of Ca and Mg supplementation in regulating maize kernel sugar content [31].

### 3.3. Physiological Mechanism of Ca and Mg Supplementation in Regulating Sugar Content of Maize Kernels

In the study of the effects of Ca and Mg on maize kernel sugar content, CAT evidently played a pivotal role. The negative regulatory effect of CAT activity underscores its critical involvement in sugar content accumulation, indicating that CAT activity directly impacts sugar accumulation, irrespective of Ca and Mg supplementation. Specifically, when Ca was applied, the GA content mediated the regulation of the sucrose and soluble sugar contents in the kernel by inhibiting CAT activity [32]. Conversely, when Mg was applied, the ABA content affected the sugar content in the kernel by promoting CAT activity, highlighting distinct regulatory mechanisms. This endogenous hormonal regulation not only prompts a reevaluation of the role of Ca and Mg in crop growth but also offers novel perspectives for maize cultivation management [33].

The findings revealed that Ca and Mg indirectly regulate the sugar content of maize kernels by influencing endogenous hormone levels and antioxidant enzyme activities. This suggests that this complex interaction must be considered in fertilization and growth regulation strategies. This discovery not only broadens the research landscape in the field of physiological regulation of crop nutrition and quality but also provides guidelines for agricultural practices [34]. It encourages agricultural producers to consider the potentially significant effects of Ca and Mg fertilizers on sugar accumulation when applying them to enhance the economic and nutritional value of crops.

Comparing previous studies with the current one, we observed both similarities and differences. While previous studies have generally acknowledged the role of CAT in plant antioxidant capacity, there is a relative scarcity of research on the effects of its interaction with endogenous hormones on sugar accumulation [35]. This underscores the prospective and innovative nature of the present study, particularly in elucidating the intricate relationship between Ca and Mg and endogenous hormones. Aligning with some arguments in the literature, the present study also underscored the importance of the dynamic balance of endogenous hormones on crop physiological responses [25]. However, in terms of specific mechanisms, especially regarding how sugar accumulation is affected through the modulation of CAT activity, our study provides new insights.

Nevertheless, the study has limitations, primarily concerning the control of the experimental conditions, the representativeness of the samples, and the potential influence of the external environment on the experimental outcomes. Future research directions should focus on a more systematic experimental design, including the effects of multiple environmental factors on the actions of calpain and endogenous hormones [36]. Additionally, employing more advanced physiological and molecular biological tools to explore the regulatory factors of CAT and its synergistic effects with other antioxidant enzymes may lead to a more profound understanding of the mechanisms of sugar accumulation in crops and the nutrient application strategies [37].

## 4. Materials and Methods

### 4.1. Experimental Planning

(1) Experimental Site Location and Climate

The experimental site was located in the Field Scientific Observation and Research Station of the College of Life Sciences, Yan’an University, Yan’an City, Shaanxi Province, China. The geographic location of the station is 36°51′30″ N, 109°19′23″ E [38]. The average annual rainfall is 540 mm, mainly concentrated in July–September; the average annual temperature is 8.8 °C; the annual number of sunshine hours is 2416 h; the annual frost-free period is 143–174 days; and there is no irrigation, as it is a typical arid–farming and rain-fed agricultural area. The test area’s parent material is loess, which is widely exposed at the ground surface. This soil is relatively homogeneous, primarily composed of yellow sandy soil, and exhibits a sandy loam texture [39].

(2) Soil Characteristics

The basic physicochemical properties of the soil were as follows: pH, 8.5; organic matter, 6.36 g kg^−1^; total nitrogen, 0.83 g kg^−1^; total phosphorus, 0.57 g kg^−1^; total potassium, 18.20 g kg^−1^; effective phosphorus, 15.30 mg kg^−1^; quick-acting potassium, 141.35 mg kg^−1^ within the 0–100 cm soil layer; and uniform soil fertility [40].

(3) Test Material

“Haoshan 168”, which is a disease–resistant, high-yielding, highly adaptable variety, with fast kernel dewatering, was selected as the spring maize variety for the test [18].

(4) Test design and treatment

Before maize planting, random replicated sampling and measurements in the field of the experimental area showed that the average effective state of Ca in the 0–40 cm soil layer of the experimental sample plot was 1208.57 mg kg^−1^, and the effective state of Mg was 117.43 mg kg^−1^, which are far below the lower limit of the threshold required for the normal growth of maize (the minimum content of Ca and Mg in maize is 5000 mg kg^−1^ and 2000 mg kg^−1^, respectively), indicating that the Ca and Mg nutrients in the maize were extremely low [4]. Therefore, a one-factor randomized experiment in our study was designed to assess the impact of varying the Ca and Mg fertilization levels on maize growth. During the entire reproductive period of maize, the experiment utilized two Ca levels—low (24.50 kg hm^−2^) and high (49.00 kg hm^−2^)—and two Mg levels—low (17.50 kg hm^−2^) and high (35.00 kg hm^−2^); the forms of Ca and Mg were substitutional and water-soluble. These levels were determined based on the deficiency standard of maize and the research of previous researchers, resulting in a total of five Ca and Mg fertilization levels [9]. The treatment, including a control group, was repeated three times across 15 experimental sample plots that measured 6 m × 6 m with a 4 m spacing between plots and 5 m protected rows between groups.

**Figure 7:**
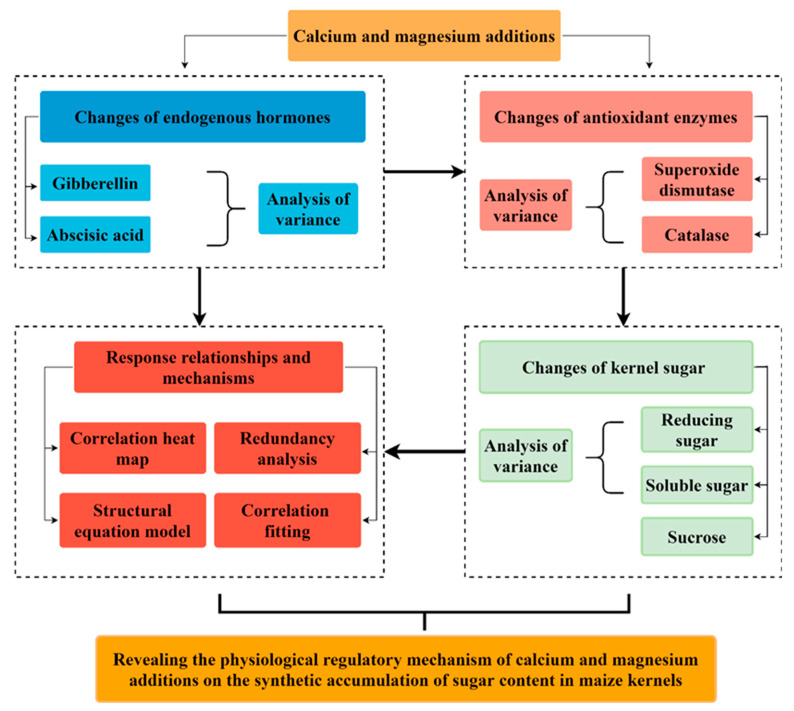
Technical scheme of this study.

The specific treatments were as follows: ① low Ca (T1); ② high Ca (T2); ③ low Mg (T3); ④ high Mg (T4); and ⑤ no Ca and no Mg (control, CK). To ensure a high efficiency and environmental safety, the Ca and Mg fertilizers were chelated with a sugar alcohol (Sugar alcohols, known as polyols, can form stable sugar–alcohol complexes with a variety of nutrients. These complexes serve as carriers for nutrient absorption, thereby enhancing the efficiency of crop nutrient uptake.). The Ca chelated with sugar alcohols had a concentration of ≥180 g L^−1^, with 250 g per bottle, and the Mg chelated with sugar alcohols had a concentration of ≥120 g L^−1^, with 300 g per bottle. These chelated fertilizers are known for their high absorption rates and green, non-toxic properties [41]. The technical scheme of this research is shown in Figure 7.

(5) Cultivation Method and Physical Dimensions

All the experimental conditions utilized monopoly furrow cultivation technology, where maize was planted on a ditch surface at a density of 60,000 hm^−2^. Each sample square featured monopoly furrows with a width of 20 cm and a height of 15 cm, with the sides of the monopoly being 10 cm in width. The furrows were separated by ditches measuring 30 cm in width, with maize plants spaced 34 cm apart within each furrow and a 50 cm interval between successive rows [11]. Maize was planted on May 10 and harvested on 12 October 2023.

(6) Fertilization and Sample Collection

Before sowing, all the experimental sample squares received an even spread of a basal fertilizer in accordance with the local recommendations for saving water and obtaining stable yields for maize, with applications of N (130 kg hm^−2^), P_2_O_5_ (120 kg hm^−2^), and K_2_O (38 kg hm^−2^) [42]. During the ripening stage, Ca and Mg fertilizers were applied at a ratio of 1:2:3:4 of the total amounts on the 1st (August 20), 10th (August 30), 20th (September 8), and 30th (September 19) days of the milky ripening stage, creating four fertilization gradients from the milky ripening stage to maturity stage. Leaves and kernels were collected on the 10th (August 30), 20th (September 8), 30th (September 19), and 40th (September 28) days after fertilization with Ca and Mg. Foliar fertilizers were selected after 16:00 h on windless and sunny days and uniformly sprayed onto maize leaves, stems, kernels, and other aboveground growth points to ensure full nutrient absorption [2].

### 4.2. Index Measurement and Methods

#### 4.2.1. Test Sample Collection

On the 10th, 20th, 30th, and 40th days of the milk ripening period of maize, 20 g samples of representative stems, functional leaves, and kernels (tasseled and threshed) were randomly collected from six maize plants with good growth and no disease in each of the experimental sample squares. This sampling was performed four times in each sample square, providing a total of 24 maize plants for the test. The samples were then mixed separately, labeled, and tightly wrapped with tinfoil, clean gauze, and aluminum foil tape [43]. They were immediately placed in a liquid nitrogen tank for freezing and promptly transported back to the laboratory for storage in an ultra-low-temperature refrigerator at −80 °C, serving as samples for testing and stored backups.

Subsequently, indoor biochemical experiments were conducted to determine the following major physiological and biochemical indicators: ① maize endogenous hormone indicators and ② maize key enzyme indicators. Concurrently, random samples of four fresh, normal maize kernels (10 g each) were collected (four repetitions for a total of 16 maize kernels). These samples were mixed, sealed in appliances to retain freshness (refrigerated ice boxes), and promptly brought back to the laboratory. After natural drying to achieve a sample moisture content of 14% ± 1%, the samples were crushed and sieved through a 40-mesh sieve for preservation as test samples, in order to determine the nutritional quality of the maize.

#### 4.2.2. Determination Methods

(1) Leaf ABA and GA contents determined using the high-performance liquid chromatography (HPLC) method [44]

For the single determination procedure, we accurately weighed 5 g (accurate to 0.001 g) of crushed maize leaves into a 100 mL volumetric flask, ensuring that the weight was precise to a thousandth of a gram. We added approximately 80 mL of methanol to the flask and then subjected the mixture to ultrasonic oscillation for 20 min to facilitate extraction. Following this, we vortexed the mixture for 2 min and adjusted the volume with methanol to serve as the extraction solution.

Next, we transferred 50 mL of the extraction solution to a separate container and subjected it to rotary evaporation at 40 °C until it was dry. We redissolved the dried methanol extract with 10 mL of water after the rotary evaporation, and then used a 1 mol L^−1^ hydrochloric acid solution to adjust the pH to approximately 2. We extracted the solution with 50 mL of ethyl acetate and collected the ethyl acetate layer. We subjected the ethyl acetate layer to rotary evaporation at 40 °C until it was dry [45].

After evaporation, we dissolved the dry extract of ethyl acetate in methanol and adjusted the volume to 1 mL. We passed the solution through a 0.45 μm filter membrane to prepare it for the high–performance liquid chromatography (HPLC) analysis.

(2) Leaf SOD activity determined using the nitrogen blue tetrazolium (NBT) photoreduction method; CAT activity determined using UV spectrophotometry [46]

Enzyme Extraction Procedure: We weighed out 0.50 g samples of maize leaves. We added 2 mL of 50 mmol L^−1^ phosphate buffer (pH 7.8, containing 1% polyvinylpyrrolidone) and a small amount of quartz sand to each sample. We homogenized the samples on ice using a grinding method, and then transferred the homogenate to a 10 mL centrifuge tube, ensuring that it was combined with the rinsing liquid. We adjusted the volume to 10 mL.

We extracted 5 mL of the solution at 4 °C and washed it to remove any remaining liquid from the sample. We centrifuged 5 mL of the extract at 10,000 rpm for 15 min at 4 °C. We collected the supernatant, which was the enzyme extract used for the determination of SOD activity and CAT activity.

Immediately following the extraction of the enzyme solution, we determined and calculated its activity and content using enzymatic reactions and a UV spectrophotometer. It is important to note the following: SOD activity was expressed as one enzyme activity unit (U), defined as the inhibit the NBT photoreduction reaction by 50%, and CAT activity was quantified in enzyme activity units (U), with one unit defined as the amount of enzyme necessary to decrease the A240 absorbance value (calibrated with distilled water) by 0.10 within a 1−min interval [47].

The specific formulas for calculating the SOD and CAT activities were as follows:

(1) $$SOD\;activity=\frac{\left(A_{0}-A_{s}\right)\times V_{t}\times 60}{A_{0}\times 0.5\times FW\times V_{s}\times t}$$

where $A_{0}$ is the absorbance of the control tube under the light; $A_{5}$ is the absorbance of the sample tube; FW is the fresh weight of the sample (g); $V_{t}$ is the total volume of the enzyme extracted from the sample (mL); $V_{s}$ is the volume of the enzyme extracted during the measurement (mL); and t is the light duration (min)

(2) $$CAT\;activity=\frac{\Delta A_{240}\times V_{t}}{0.1\times V_{s}\times t\times FW}$$

where $\Delta A_{240}=A_{S0}-\frac{A_{S1}+A_{S2}}{2}$; $A_{s0}$ is the absorbance of the control tube of the boiled enzyme solution; $A_{s1}\;and\;A_{s2}$ are the absorbances of the sample tube; V_t_ is the total volume of the enzyme extract (mL); Vs is the volume of the enzyme extract (mL) at the time of measurement; and FW is the fresh weight of the sample (g).

(3) Kernel sugar content determined using the anthrone colorimetric method [48]

We weighed 50 mg of the maize kernel powder sample into a 10 mL graduated centrifuge tube. We added 4 mL of 80% ethanol to the tube and placed it in a water bath at 80 °C for 40 min. After this period, we centrifuged the mixture and collected the supernatant. Next, we added 10 mg of activated carbon to the supernatant and decolorized it at 80 °C for 30 min. We brought the volume up to 10 mL with 80% ethanol, and then filtered the solution. We transferred 1 mL of the filtrate into a stoppered test tube and added 5 mL of the anthrone reagent. We mixed the contents thoroughly and heated the mixture in a boiling water bath for 10 min. We removed the test tube immediately and cooled it in water to 25 °C. Finally, we used a visible spectrophotometer to determine and calculate the content of soluble sugars, sucrose, and reducing sugars using the appropriate formula [49].

(3) $$\mathrm{Sugar}\;\mathrm{content}\;(\%)=\frac{S\times V_{2}\times V_{3}\times 100}{V_{1}\times V_{4}\times m\times 10^{6}}$$

where S is the amount of sugar in the sample liquid to be measured (μg); m is the weight of the sample (g); V_1_ is the volume of the diluted sample used for determination (mL); V_2_ is the total volume of the diluted sample used for analysis (mL); V_3_ is the total volume of the sample solution (mL); and V_4_ is the volume of the sample liquid used for hydrolysis (mL).

### 4.3. Statistical Methods

Analysis of variance (ANOVA) was conducted to assess the significance of differences in endogenous hormone levels and antioxidant enzyme activities in maize leaves at the various maturity stages under the different Ca and Mg supplementation levels. Heatmaps were employed to illustrate the patterns of sugar content variation in the maize kernels across the different Ca and Mg supplementation levels, from the milky stage to full maturity. Correlation heatmaps were utilized to visualize the intrinsic relationships among endogenous hormones, antioxidant enzymes, and sugars. Redundancy analysis and structural equation modeling were applied to elucidate the differences in the sugar content of maize kernels under the varying Ca and Mg supplementation levels. Through these analyses, we identified the core physiological regulators and the underlying pathways that drive sugar content changes in maize kernels following the addition of Ca and Mg.

## 5. Conclusions

This study elucidated the physiological effects of Ca and Mg on maize leaves and kernel quality by assessing antioxidant enzyme activities, endogenous hormone levels, and sugar contents under varying Ca and Mg conditions. The key findings were as follows:

(1) The high−Mg treatment significantly increased the SOD activity and antioxidant capacity in maize leaves, while the low−Ca and Mg treatments resulted in lower SOD activity with limited effects. The high−Ca treatment was the most effective in enhancing CAT activity during the milk ripening to ripening stage. The low−Ca treatments promoted GA synthesis at the late ripening stage, whereas the high−Ca and Mg treatments inhibited GA at the early ripening stage. The high−Mg treatments also increased the ABA content, potentially enhancing stress tolerance, while the low−Ca treatments had no significant effect on the ABA content.
(2) The high−Ca treatment was the most effective in enhancing the RS content in maize kernels, while the low−Mg treatment was the most effective in increasing the SU content. Both the low- and high−Mg treatments increased the SS content, indicating the importance of Mg for the energy supply.
(3) The core physiological pathway regulating the sugar content in maize kernels after Ca supplementation was “GA−CAT−SU and SS”, with GA negatively regulating CAT activity, which in turn, negatively regulated the SU and SS contents. After Mg supplementation, the pathway was “ABA−CAT−SU and SS”, with ABA positively regulating CAT activity, which then negatively regulated the SU and SS contents. In the control treatments, endogenous hormones affected CAT activity, but the regulation by GA and ABA was opposite to that observed with Ca and Mg supplementation. The relationship between endogenous hormones and the maize kernel sugar content was complex, exhibiting a quadratic nonlinear relationship rather than a simple linear one.

These results highlighted the differential effects of Ca and Mg on maize physiology and provided insights into their roles in regulating the kernel sugar content through endogenous hormones and antioxidant enzymes. These findings not only enhance our understanding of the role of micronutrients in maize growth but also provide new strategies for improving maize quality and stress tolerance.

## Data Availability

The data that support the findings of this study are available from the corresponding author upon reasonable request.
